# Open Evaluation: A Vision for Entirely Transparent Post-Publication Peer Review and Rating for Science

**DOI:** 10.3389/fncom.2012.00079

**Published:** 2012-10-17

**Authors:** Nikolaus Kriegeskorte

**Affiliations:** ^1^Medical Research Council, Cognition and Brain Sciences UnitCambridge, UK

**Keywords:** peer review, publishing, ratings, social web, open evaluation

## Abstract

The two major functions of a scientific publishing system are to provide *access* to and *evaluation* of scientific papers. While open access (OA) is becoming a reality, open evaluation (OE), the other side of the coin, has received less attention. Evaluation steers the attention of the scientific community and thus the very course of science. It also influences the use of scientific findings in public policy. The current system of scientific publishing provides only journal prestige as an indication of the quality of new papers and relies on a non-transparent and noisy pre-publication peer-review process, which delays publication by many months on average. Here I propose an OE system, in which papers are evaluated post-publication in an ongoing fashion by means of open peer review and rating. Through signed ratings and reviews, scientists steer the attention of their field and build their reputation. Reviewers are motivated to be objective, because low-quality or self-serving signed evaluations will negatively impact their reputation. A core feature of this proposal is a division of powers between the accumulation of evaluative evidence and the analysis of this evidence by paper evaluation functions (PEFs). PEFs can be freely defined by individuals or groups (e.g., scientific societies) and provide a plurality of perspectives on the scientific literature. Simple PEFs will use averages of ratings, weighting reviewers (e.g., by *H*-index), and rating scales (e.g., by relevance to a decision process) in different ways. Complex PEFs will use advanced statistical techniques to infer the quality of a paper. Papers with initially promising ratings will be more deeply evaluated. The continual refinement of PEFs in response to attempts by individuals to influence evaluations in their own favor will make the system ungameable. OA and OE together have the power to revolutionize scientific publishing and usher in a new culture of transparency, constructive criticism, and collaboration.

## Introduction

A scientific publication system needs to provide two basic functions: access and evaluation. Access means we can read anything, evaluation means we do not have to read everything. The traditional publication system restricts the access to papers by requiring payment, and it restricts the evaluation of papers by relying on just 2–4 pre-publication peer reviews and by keeping the reviews secret. As a result, the current system suffers from a lack of quality and transparency of the peer-review evaluation process, and the only immediately available indication of a new paper’s quality is the prestige of the journal it appeared in.

Open access (OA) is now widely accepted as desirable and is in the process of becoming a reality (Harnad, 2010). However, the other essential element, evaluation, has received less attention. The current peer-review system has attracted much criticism (Smith, 2006, 2009; Ware, 2011). Arguments (Smith, 1999; Godlee, 2002; Frishauf, 2009; Boldt, 2010) and experiments (Harnad, 1997; Walsh et al., 2000; Greaves et al., 2006; Pulverer, 2010; Pöschl, 2010) with open review and post-publication commentary have suggested that a more transparent system might have potential. However, we have yet to develop a coherent shared vision for “open evaluation” (OE), and an OE movement comparable to the OA movement.

The evaluation system steers the attention of the scientific community and, thus, the very course of science. For better or worse, the most visible papers determine the direction of each field and guide funding and public policy decisions. Evaluation, therefore, is at the heart of the entire endeavor of science. As the number of scientific publications explodes, evaluation and selection will only gain importance. A grand challenge of our time, therefore, is to design the future system, by which we evaluate papers and decide which ones deserve broad attention. OE, an ongoing post-publication process of transparent peer evaluation (including written reviews and ratings of papers), promises to address the problems of the current system.

Here I outline a vision for an open publication and evaluation system with the following key features: Papers are evaluated in an ongoing fashion after publication by means of reviews and ratings. Reviews are mini-publications and can be signed or anonymous. Signed reviews and signed ratings both contribute to a scientist’s visibility. More important papers are more deeply evaluated as they will receive more evaluations. Scientists are more motivated to perform reviews, because it helps build their reputation. Multiple paper evaluation functions (PEFs), freely defined by individuals or groups (e.g., scientific societies, private, and public organizations) provide a plurality of perspectives on the scientific literature. The transition toward a future system of instant publication can be achieved by providing an OE system that will initially serve to more deeply evaluate important papers published under the current system of pre-publication peer review. When the OE system has proven its superiority to the current system of peer review, it will replace the current system.

First, I briefly describe key features of the current system of scientific publishing and where it falls short. Second, I briefly describe some positive current developments that represent steps in the right direction, but do not go far enough. Third, I present a general vision for scientific publishing, based on OA and OE, using entirely transparent post-publication reviews and ratings and freely definable PEFs. Fourth, I describe a specific plan for a minimalist OE system that is simple and yet could go a long way toward providing the key functionality for accumulating the evaluative evidence. Fifth, I describe a specific plan for a PEF, so as to illustrate more concretely how the accumulated evidence can be combined to prioritize the literature. Sixth, I outline the ultimate goal, free instant scientific publishing with OA and OE. Finally, in the discussion, I address a number of concerns and counterarguments that have frequently come up in informal discussions. These concerns include a lack of evaluations and the question of how we might smoothly transition toward the envisioned system.

## The Current System of Scientific Publishing

**Figure 1:**
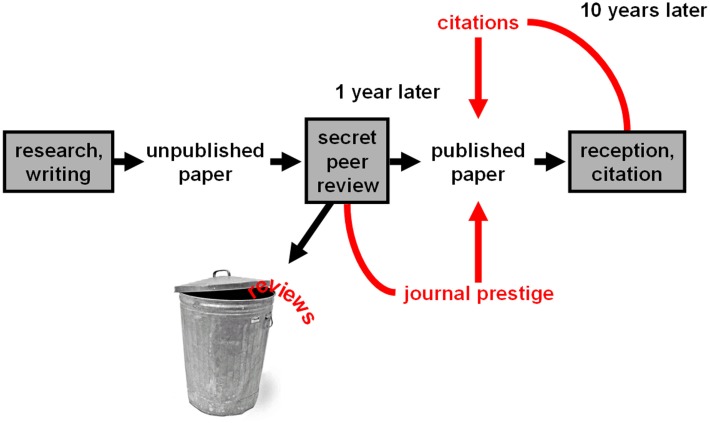
**The current system**. This flowchart summarizes the process by which the current system operates. Key features include long publication delays, secret peer review, failure to make evaluations (reviews and ratings) available to the community, and journal prestige as the only evaluative signal available immediately upon publication.

The current system of scientific publishing provides access and evaluation in a limited fashion. While access often requires payment, papers are made available in an appealing professional layout that makes them easier to read. This function is desirable, but not critical to scientific progress. The current system also provides evaluation: It administers peer review and provides an evaluative signal that helps readers choose papers, namely journal prestige. This function is critical to scientific progress. However, journal prestige is a crude measure that is not specific to particular papers. The overall process of the current system is summarized in Figure 1. We will now discuss the main drawbacks.

### The system is not generally open access

Scientific papers benefit society only to the extent that they are accessible. If the public pays for scientific research it should demand that the results be openly accessible. If private publishers offer valuable services at reasonable prices that contribute to the dissemination of scientific papers, such as appealing layout, then research institutes may want to purchase them. However, access to results of publicly funded research should never come at a cost to an individual. Since OA is already widely seen as desirable among scientists and the general public, this paper focuses on OE: how to open up the other major function of a publication system, namely the evaluation of scientific papers.

### Journal prestige, the only quality indicator for new papers, provides an impoverished and unreliable evaluative signal

The main evaluative signal provided to readers for prioritizing their reading of scientific papers is journal prestige. We are more likely to attend to a paper published in *Nature* than to a similar paper published in a specialized journal. While journal prestige is somewhat correlated with the quality of scientific papers, it is not a reliable indicator of the quality of a particular paper. Moreover, journal prestige as an evaluative signal is compromised by causal circularity: Prestige – once acquired – creates its own reality.

**Figure 2:**
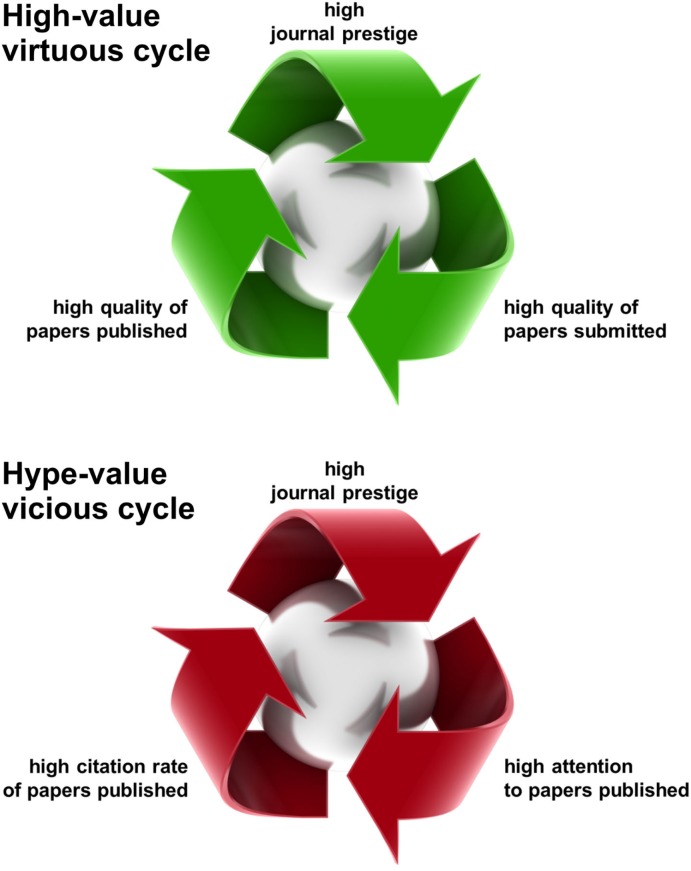
**The self-fulfilling prophecy of journal prestige**. Journal prestige creates its own reality through two types of self-fulfilling prophecy. The first type (top) is a virtuous cycle, in which high prestige leads to high-quality submissions. As a result, the papers selected for publication are also of higher quality, which in turn contributes to journal prestige. This cycle is virtuous because it makes journal prestige a somewhat reliable signal. The second type of self-fulfilling prophecy (bottom) is a vicious cycle, in which high prestige leads to high attention being paid to papers (even those of lesser quality). This boosts their citation rate, which in turn contributes to journal prestige. This cycle is vicious because it compromises the reliability of journal prestige as an evaluative signal. In the current system, journal prestige is the only evaluative signal available for new papers, so it is widely relied upon by scientists, despite its limited reliability.

The self-fulfilling prophecy of journal prestige has two component cycles of causality, a virtuous one and a vicious one (Figure 2). In the virtuous cycle, prestige brings higher-quality submissions, which in turn contribute to prestige. This cycle is virtuous, because the increase in prestige actually reflects an increase in the quality of the papers. In the vicious cycle, prestige brings higher-citation frequencies (even for average-quality papers), which in turn brings prestige. This cycle is vicious, because it causes journal impact factors (IF) to give a distorted picture of the quality of the published papers. IFs and the higher or lesser prestige they confer on journals therefore compromise the public perception of the quality of particular papers.

In addition to being an unreliable indicator of a scientific paper’s quality, journal prestige provides only a greatly impoverished, evaluative signal. The detailed reviews and multi-dimensional ratings provided to the journal by the reviewers are kept secret. The reviewers are established experts, largely funded by the public, who work hard to evaluate scientific papers. And yet the detailed evaluations are kept secret and contribute to the reception of a paper only after being reduced to a categorical quality stamp: the journal label. This constitutes a loss to the scientific community and to the general public of valuable judgments that are already being performed and paid for.

### The review process is non-transparent, time-limited, and based on too few opinions

The current system of publishing is based on a non-transparent evaluation process that includes secret reviews visible only to editors and authors. For high-impact publications, the editorial decision process preceding full review often also includes informal comments solicited by the editors from other scientists. Such informal additional sources of evaluation may often improve the quality of the decisions made – this is why they are used. Nevertheless, this practice compromises the transparency and objectivity of the system.

The selection of a paper for publication is typically based on 2–4 peer reviews. The quality of an original and challenging scientific paper cannot reliably be assessed by such a small number of reviewers – even if the reviewers are experts and have no conflict of interest (i.e., they are not competitors). In reality, the reviewers who are experts in the particular topic of a paper often have some personal stake in the paper’s publication. They may be invested in the theory supported or in another theory. More generally, they may have competitive feelings that compromise their objectivity.

For high-impact publications, this political dynamic is exacerbated because the stakes are higher and more scientists are competing for a smaller stage. To make matters worse, high-impact publications require their reviewers to judge the significance of the paper. Judging a paper’s significance requires a necessarily somewhat subjective projection as to where the field will move and how it will be affected by the paper under review. Despite these additional sources of noise in the value signal provided by the reviews, high-impact journals – more than specialized journals – need *precise* quality assessments if they are to realize their claim of selecting only the very best papers.

### Authors and reviewers operate under unhealthy incentives

Even if the majority of scientists are principally motivated by a desire to find the truth and maintain a high level of personal ethics, the incentives built into the system influence the level of objectivity achieved in the writing of papers and in the evaluation process. The current system provides several unhealthy incentives:

- It rewards authors for making claims that are stronger than can be justified (as this increases the chances of selection by editors for publication in high-impact journals).
- It rewards authors for suggesting reviewers known to be friendly or supportive of the claims and for selectively citing other scientists likely to support publication (as these are more likely to be selected as reviewers).
- It rewards reviewers for spending little time reviewing (as this is time available for their own science and reviewing is not rewarded or even recorded). This encourages reviewers to decline many reviews and to avoid in-depth evaluation of the ones they accept.
- It rewards reviewers for obstructing or delaying the publications by competitors and for expediting publications by allies.

Most scientists may resist these rewards. However, an ideal system would not provide such unhealthy incentives. To obstruct or expedite publication, a reviewer need not make any false statements, but merely to gage the review’s level of enthusiasm and focus on strengths or weaknesses as needed. Since the reviews and the reviewer’s identity are kept secret, there is no public scrutiny of either the arguments in a review or possible conflicts of interest of the reviewer. A rogue reviewer can therefore act with impunity and distort decisions indefinitely. The antidote to corruption is transparency – this is a central motivation for the present proposal.

### Evaluation delays publication

The current system of journal-controlled pre-publication review delays publication of papers by months in the best case. When authors target prestigious journals, multiple rejections and rounds of review and revision, often delay publication by more than a year from the date of initial submission. Scientific papers are the major mode of formal scientific communication. Delays of many months in this crucial communication line slowdown the progress of science.

### The system is controlled by for-profit publishers and incurs excessive costs

In the current system, the key function of evaluating and selecting papers is controlled by private publishing companies. Although papers are reviewed by scientists, the selection of reviewers and the decisions about publication are largely in the hands of private publishers. The publishers are professional at what they do, draw from a large amount of experience, and have a reputation to defend. However, profit maximization can be in conflict with what is best for science. The current system is immensely profitable to the publishers, so they are not natural leaders of a transition to a better system. More generally, the arguments in favor of direct public funding of not-for-profit research institutes (as opposed to buying studies from private research institutes) also apply to scientific publishing. To the extent that the free market can provide cost-efficient solutions, there is a place for the private sector. However, we need to assess whether the benefit to science of the services provided justifies the cost of the current system.

## Some Recent Positive Developments

Many positive developments in scientific publishing include the Public Library of Science (PLoS) and other open-access journals, the Frontiers journals, Faculty of 1000, and ResearchBlogging.org^1^. In this section we briefly describe each of these developments and explain why they represent important steps in the right direction, but do not go far enough to fully address the problems related to the way the current system utilizes peer review.

### Public library of science

The PLoS journals^2^ combine OA with beautiful professional layout and well-designed web-interfaces. Moreover, the websites offer functionality for post-publication commentary and 1–5-star ratings on three scales (“insight,” “reliability,” and “style”) for registered users. Every paper has a “metrics” page that shows these ratings, along with usage statistics (views, pdf downloads), citation counts from multiple sources, and social-network links. The presence of these features is exemplary. PLoS ONE^3^ takes a further step forward by using pre-publication review only to establish that a paper is “technically sound,” not to assess its importance. This is likely to render peer review more objective. The PLoS journals combine a high scientific standards and high production value with OA.

Although the post-publication commentary and rating functionalities represent an attempt at integrating OE, these features are not widely used and thus do not yet provide a major evaluative signal at the moment. This highlights the challenge to motivate scientists to contribute to post-publication evaluation. The PLoS family of journals relies on the traditional process of secret pre-publication peer review as the core of its evaluation process. In a fully transparent post-publication system as proposed here, the editor-solicited initial reviews and ratings would be public, so every paper would have multiple reviews and ratings from this process. For specialized papers, such as those published in PLoS ONE, it is not realistic to expect many additional reviews to accumulate. Moreover, commenting on PLoS papers requires login (increasing the effort required), but comments and ratings are not part of the core evaluation mechanism (which remains secret pre-publication peer review). A scientist who might want to share an opinion has minimal motivation to use the commenting system because there is little indication that such a contribution will matter as the paper already has its mark of approval from pre-publication peer review. A signed critical comment, in particular, would mean taking a social risk without promising much positive impact. As we will see below, the change of culture required to make a transparent evaluation system work requires that the post-publication evaluations really matter as more than an add-on and that signed reviews count as mini-publications that are citable and help build the reviewer’s reputation.

### The Frontiers journals

The Frontiers journals^4^, starting with Frontiers in Neuroscience, combine OA, a new system for constructive and interactive pre-publication peer review, web-based community tools, and post-publication quasi-democratic evaluation of papers. Moreover, Frontiers provides a hierarchy of journals from specialized (e.g., Frontiers in Computational Neuroscience) to general (Frontiers in Neuroscience). The hierarchy may be extended upward in the future.

Importantly, papers are first published in the specialized journals. Based on the additional evaluative information accumulated in the reception of the papers by the community, a subset of projects is selected for wider publication in a higher-tier journal. This has several advantages over conventional approaches: Selection for greater visibility is based on more evidence than available to traditional high-impact publications (which rely only on the few reviews and informal opinions they solicit). The higher-tier thus responds more slowly and ideally more wisely: avoiding to draw attention to findings that do not pass the test of confrontation with a larger group of peer scientists than can be asked to initially review a paper. Like PLoS, Frontiers offers web functionality for reviewing and rating, but these OE features do not yet form the core of the evaluation process.

The Frontiers system is visionary and represents a substantial step in the right direction. As for the PLoS journals, however, quality control for the lowest tier still relies on pre-publication review, tolerating the evaluation inaccuracies and delays and failing to provide detailed evaluative information, such as public reviews.

### Faculty of 1000

Faculty of 1000^5^ provides very brief post-publication recommendations of papers with a simple rating (“Recommended,” “Must-read,” “Exceptional”). The post-publication review idea is a step forward. However, the reviewing is limited to a select group of highly distinguished scientists – a potential source of bias. Evaluations are recommendations – there is no mechanism for negative reviews. Numerical evaluations are unidimensional thus providing only a very limited signal. Finally, the recommendation text is a brief statement, not a detailed review. In addition, the Faculty of 1000 system is a for-profit effort that is not designed or controlled by the scientific community. It is post-publication peer evaluation, but it is not OE. And it is not OA, either: The evaluations are sold by subscription.

### ResearchBlogging.org

ResearchBlogging.org collects blog-based responses to peer-reviewed papers. This is a big advance as it allows anyone to participate and provide evaluative information, which can be accessed through the ResearchBlogging.org website. The use of blogs is helpful in that it makes this system open. However, it also means that reviews are not permanently citable as blogs can be taken down. Moreover, as of yet the blog responses lack numerical ratings that could be automatically analyzed for paper evaluations. Blog responses are also not digitally signed for author identification, and the responses are not visible when viewing the target paper itself.

## A Vision for Open Evaluation

**Figure 3:**
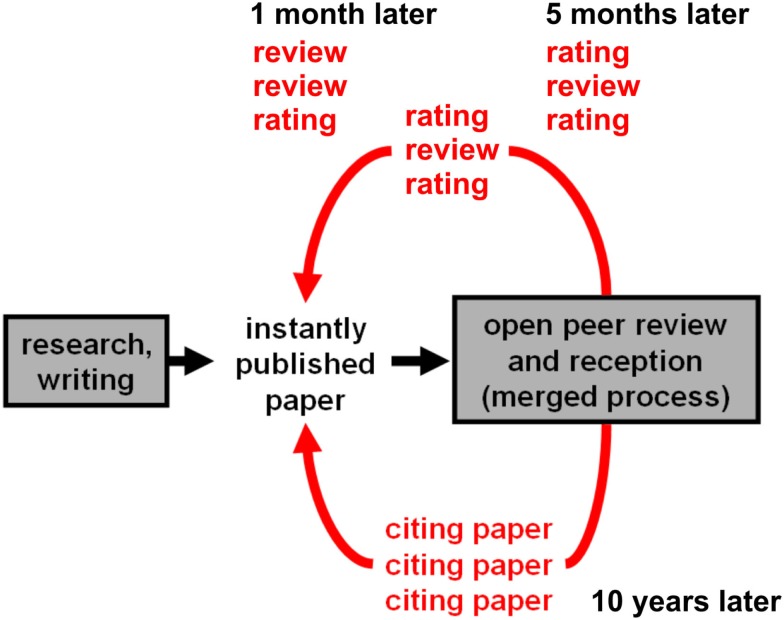
**The future system**. This flowchart summarizes the process by which the future system will operate. Key features include instant publication, open peer review, published evaluations (reviews and ratings) enabling the community to evaluate the evaluations and form their own judgment, and ongoing accumulation of evaluative evidence after publication.

**Figure 4:**
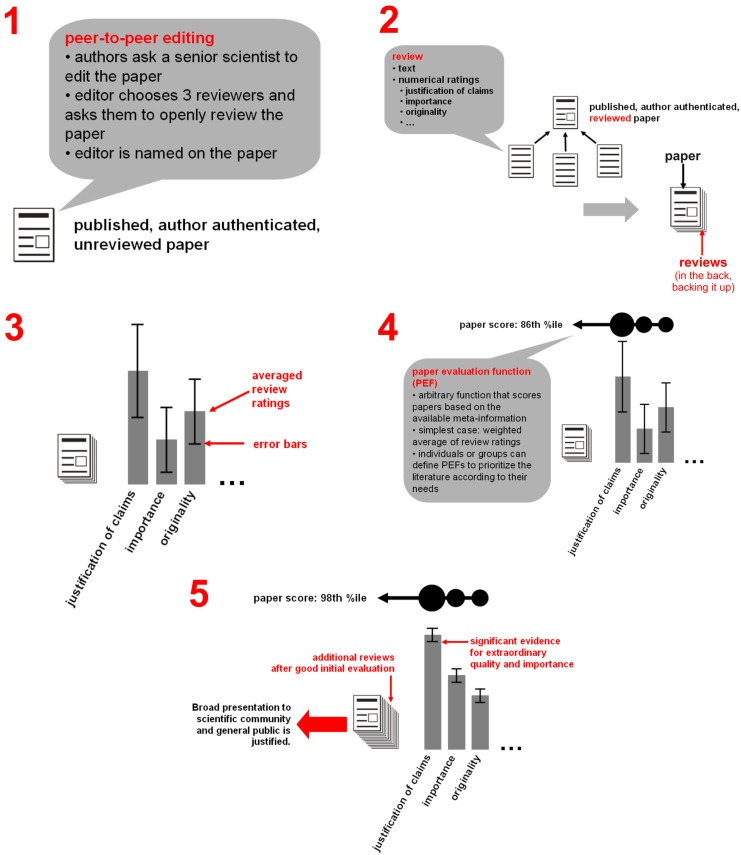
**Step-by-step overview of the proposed process of open publication and evaluation**. (1) The paper is instantly published to the entire community. Reception and reviewing commence. Although anyone can review the paper, peer-to-peer editing can help encourage a balanced set of reviewers to get the process started. (2) Reviews and ratings are linked to the paper. They need not be viewed, but are present in the back, “backing up” its claims. (3) Rating averages can be viewed with error bars that tend to shrink as ratings accumulate. (4) Paper evaluation functions (PEFs) can be arbitrarily defined to combine a paper’s evaluative information into a single score. PEFs can be simple, e.g., a weighted average of ratings, where weights can depend on the rating scale (e.g., justification of claims versus importance) or on reviewer information (e.g., well-published senior scientist versus student). PEFs can also be complex, e.g., a Bayesian inference algorithm jointly evaluating the claims of an entire field’s network of papers. (5) The evaluation process is ongoing. In case the paper score rises to a very high percentile with high confidence, presentation to a broad audience is justified.

The problems of the current system can all be addressed by open post-publication peer review. The basic process of this model is summarized in Figure 3 and illustrated in greater detail in Figure 4.

### Open

Any scientist can instantly publish a peer review on any published paper. The scientist will submit the review to a public repository (see also Florian, 2012 in this collection). Reviews can include written text, Figures, and numerical quality ratings on multiple scales. The repository will link each paper to all its reviews, such that readers can readily access the evaluative meta-information whenever they view a paper. Peer review is open in both directions: (1) Any scientist can freely submit a review on any paper. (2) Anyone can freely access any review as soon as it is posted.

### Post-publication

Evaluations are posted after publication, because a paper needs to be publicly accessible in order for any scientist to be able to review it. Post-publication reviews can add evaluative information to papers published in the current system (which have already been secretly reviewed before publication). For example, a controversial paper appearing in *Science* may motivate a number of supportive and critical post-publication reviews. The overall evaluation from these public reviews will affect the attention given to the paper by potential readers. The written reviews may help readers better understand and judge the paper.

### Peer reviews

Like the current system of pre-publication evaluation, the new system relies on peer reviews and ratings. For all of its faults, peer review is the best mechanism available for evaluation of scientific papers. Note however, that public post-publication reviews differ in two crucial respects:

(1) They do not decide about publication – as the papers reviewed are already published.
(2) They are public communications to the community at large, not secret communications to editors and authors.

This makes the peer review more similar to getting up to comment on a talk presented at a conference. Because the reviews are transparent and do not decide about publication, they are less affected by politics. Because they are communications to the community, their power depends on how compelling their arguments are to the community. This is in contrast to secret peer review, where uncompelling arguments can prevent publication because editors largely rely on reviewers’ judgments and reviewers are not acting publicly before the eyes of the community.

### Peer ratings

The term “evaluation” refers to both reviews and ratings. Like peer reviews, peer ratings are used by many journals in the current system. However, the valuable multi-dimensional quantitative information they provide remain secret. The OE system will enable explicit ratings on multiple scales that reflect both the confidence that the claims are veridical and the importance of the paper. Scales will include “justification of claims,” “novelty of claims,” and “significance of claims.” The system will also include a simple syntax for freely introducing new scales within any evaluation. All this requires is to give the new scale a name that clearly denotes its meaning and to provide a rating.

### Multiple lenses onto the literature

**Figure 5:**
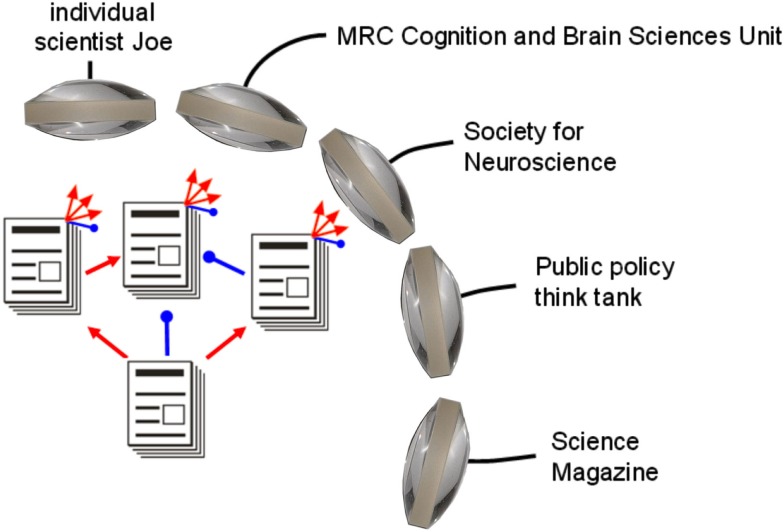
**A plurality of paper evaluation functions (PEFs) provides multiple lenses onto the literature**. Organizations and individuals can define PEFs according to their own priorities and make the resulting paper rankings publicly available. Competing PEFs provide multiple perspectives. Moreover, the OE system becomes “ungameable” as PEFs respond to any attempts by individual scientists or groups to take advantage of weaknesses of current PEFs. With constantly evolving PEFs, each scientist and organization is motivated to aim for truth and objectivity. Red and blue pointers correspond to “excitatory” and “inhibitory” evaluative links, which could be represented by positive and negative numerical ratings. Beyond simple averaging of ratings, PEFs could employ sophisticated inference algorithms to jointly estimate the probabilities of all papers’ title claims.

The necessary selection of papers for reading can be based on the reviews and their associated numerical judgments. Any group or individual can define a PEF based on content and quality criteria. A PEF could for example, rely only on signed ratings from post-PhD scientists, and weight different rating scales in a particular way. A PEF could also employ social-network information, e.g., to downweight ratings from reviewers that are associated with the authors. Social networks could also contribute evaluative information on papers to PEFs, including usage and sharing statistics as well as ratings (Lee, 2012; Priem and Hemminger, 2012; Walther and van den Bosch, 2012; Zimmermann et al., 2012; all in this collection). Beyond weighted averaging, PEFs could use complex recurrent inference algorithms, e.g., to infer probabilities for the title claims of papers. Social web and collaborative filtering algorithms (Goldberg, 1992; Breese et al., 1998; Schafer et al., 2007) will be applied to this problem. However, evaluating the scientific literature poses unique challenges and requires greater transparency and justification than product recommendation systems. The development of PEFs will build on and extend the existing literature on collaborative filtering systems. There will be a plurality of PEFs prioritizing the literature from multiple perspectives (Figure 5). When reviewers start using a new rating scale in their evaluations, PEFs may utilize the ratings on the new scale if the evaluative evidence the scale provides is thought to justify its inclusion.

### Web-portals as entry points to the literature

Web-portals (“subject focal points,” Smith, 1999) will serve as entry points to the literature, analyzing the numerical judgments in the reviews by different criteria of quality and content (including the use of meta-information about the scientists that submitted the reviews). There will be many competing definitions of quality – a unique one for each web-portal and each individual defining his or her own PEF. Web-portals can define PEFs for subcommunities – for scientists too busy to define their own. A web-portal can be established cheaply by individuals or groups whose members share a common set of criteria for paper prioritization.

### Merging review and reception

Currently review is a time-limited pre-publication process and reception of a paper by the community occurs later and over a much longer period, providing a very delayed – but ultimately important – evaluative signal: the number of citations a paper receives. Open post-publication peer review will remove the artificial and unnecessary separation of review and reception. It will provide for a single integrated process of open-ended reception and review of each paper by the community. Important papers will accumulate a solid set of evaluations and bubble up in the process – some of them rapidly others after years.

### Signed and anonymous evaluations

There is some evidence that the threat of revealing the reviewer’s identity to the authors (van Rooyen et al., 1999) or of making a review public (van Rooyen et al., 2010) may just deter reviewers and do little to improve review quality. This highlights the need to give reviewers a choice of whether or not to sign. Moreover, defining reviews as open letters and mini-publications will create a different culture, in which scientists define themselves not only through their own work, but also through others’ work they value. Signed evaluations have the advantage that they attach the reviewer’s reputation to the judgment, thus alleviating abuse of reviewer power (Walsh et al., 2000; Groves, 2010). Anonymous reviews have the advantage that they enable reviewers to criticize without fear of negative consequences (Khan, 2010). Both types are needed, and a scientist will make this choice on a case-by-case basis. The anonymous option will encourage communication of critical arguments. But to the extent that an argument is objective, sound, and original, a scientist will be tempted to sign in order to take credit for his or her contribution. In analyzing the review information to rank papers, signed reviews can be given greater weight if there is evidence that they are more reliable. Reviewers can digitally sign their reviews by public-key cryptography^6^. The idea of digitally signed public reviews has been developed here^7^, where further discussion and a basic software tool that implements this function can be found.

### Reviews as open letters to the community

Reviews will no longer be secret communications deciding about publication. They will be open letters to the community with numerical quality ratings that will influence a paper’s visibility. OE will initially build on the current system by providing higher-quality transparent evaluations of papers that have already been reviewed secretly before publication. As long a traditional peer review is in place, we expect mainly important papers to attract additional OEs. The original pre-publication reviewers could use the OE system to make their reviews (updated to reflect the published revision) public, so that their work in reviewing the paper can be of benefit to the readers of the paper and to the community at large.

### Improving evaluation quality

The quality of the evaluative signals will be improved by post-publication review for a number of reasons:

(1) Since reviews are open letters to the community, their power is dependent on how compelling they are to the community. (In the present system, rejecting a paper does not require an argument that would hold up under the scrutiny of the community. For a high-impact journal, for example, all it takes is to say that the paper is good, but not sufficiently surprising.)
(2) The system will include signed evaluations, so the reviewer’s reputation is on the line: he or she will want to look objective and reasonable. (Anonymous evaluations can be downweighted in assessment functions to the degree that they are thought to be unreliable.)
(3) Important papers will accumulate more evaluations (both reviews and ratings) over time as the review phase is open ended, thus providing an increasingly reliable evaluative signal.

Ratings, like reviews, can be signed and will enable us to help steer the attention of our field without investing the time required for a full review. Early signed ratings that turn out to be solid can contribute to a scientist’s reputation just as reviews can. As researchers read and discuss the literature in journal clubs around the world as needed for their own research, the expert judgments are already being performed behind closed doors. The OE system will provide a mechanism for feedback of this valuable information into the public domain. With PEFs in place to summarize the evaluations, journal prestige will eventually not be needed anymore as an evaluative signal.

### Community control of the critical function of paper evaluation

Open evaluation means that the scientific community organizes the evaluation of papers independently, thus taking control of this critical function, which is currently administered by publishers. Evaluation is the key function that currently keeps science dependent on for-profit publishers. Achieving OE, therefore, will also help accelerate the ongoing shift toward general OA. Conversely, OA is a requirement for true OE, as only openly accessible papers can be evaluated by the entire community. OA and OE are the two complementary pieces of the ongoing paradigm shift in scientific publishing.

### A division of powers between the accumulation of evaluations and the prioritization of the literature

A core feature of this proposal is a clear division of powers between the OE system, which accumulates reviews and ratings and links them to the papers they refer to, and the PEFs, which combine the evaluative evidence so as to prioritize the literature from particular perspectives. This division of powers requires that the evidence accumulated by the OE system is publicly available, so that independent groups and individuals can analyze it and provide PEFs. This division of powers ensures transparency and enables unrelated groups and individuals to freely contribute to the evaluative evidence and to its combination for prioritizing papers. For example, if a group of scientists started doing mutual favors by positively evaluating each other’s papers, an independent group could build a PEF that uses only signed evaluations and downweights evaluations from individuals within cliques of positive mutual evaluation. Conversely, when a web-portal claims to combine the evaluative evidence by a given PEF to compute its paper ranking, anyone can re-implement that algorithm, run it on the public evaluative evidence, and check the ranking for correctness. This fosters a culture in which we keep each other honest, and in which public interest and self-interest are aligned. When the process is entirely transparent and competing PEFs evolve in response to any attempts to game the system, an individual’s best bet is to act according to the criteria of objectivity he or she believes will eventually prevail.

## A Specific Plan for a Minimalist Open Evaluation System

What are the minimal requirements for a web-based OE system for accumulating evaluations? We would like the system to enable rapid ratings, signed or unsigned, and also multi-dimensional ratings and in-depth reviews. A key consideration is the time it takes for users to provide ratings as this will determine the efficiency of the system and, thus, the volume of evaluative evidence accumulated. I will now describe a prototype that meets minimum requirements and is designed to “seduce” the user to provide more detailed information.

### What kind of rating scale?

The quickest rating is clicking a “like” button. While this has proven useful for prioritizing items in non-scientific web systems, it is not ideal for evaluating scientific papers. The key argument against one-click ratings is that they provide continuous valuations only in aggregate. Counting the number of likes confounds the amount of exposure a given item (e.g., a paper) has received (how many people considered clicking “like”) with the value attributed to it. Adding a “dislike” button enables us to consider the balance of likes and dislikes. However, a continuous valuation requires a sizeable number of contributions, and error bars on the valuation require even more contributions. “Like” and “dislike” buttons, therefore, are ideal for sampling casual judgments of large numbers of people, but less suited for our present purpose, i.e., sampling careful judgments of small numbers of people.

I therefore suggest using an overall rating scale as the first evaluative piece of information. The fastest way to collect a continuous judgment might be a click on a continuous scale on the screen. However, we are interested in careful deliberate evaluations. We therefore prefer the user to decide on a numerical rating. A numerical rating is also better suited for being explicitly remembered and communicated. Entering one number takes only a little longer than a click.

The next question is how the single scale should be defined. Rating scales for movies and other cultural items sometimes use a five-star system. However, a five-level scale appears too coarse to reflect individual scientists’ quality judgments on papers and also does not provide a sufficiently fine-grained signal for prioritization entire literatures. A higher resolution appears desirable, e.g., a number between 0 and 100. Bounding the ratings between a lowest and highest value provides an intuitive definition of its units, e.g., from worst to best imaginable. Ideally, however, the units of the scale should be defined more precisely than by a mere specification of bounds. In that case bounding the scale is not necessary.

A rating could be conceptualized as a “weight,” which the rater thinks should be given to the paper in combining the evidence on a scientific question (as in optimal linear estimation). This would suggest that 0 should be the lowest possible rating. A rating of 0 would communicate the judgment that the paper’s contents are best ignored in order to arrive at the truth. Note, however, that limiting the scale to positive values entails that the average across multiple noisy ratings will be positively biased (i.e., the average will always be greater than 0 even if the paper deserves a weight of 0). To address this shortcoming, ratings could comprise negative as well as positive numbers. This possibility is illustrated in Figure 5. Positive and negative ratings could provide excitatory and inhibitory connections in an evaluative network. This would enable negative judgments to balance positive judgments and reduce the effective weight given to a paper (as estimated by an average across the ratings) all the way to 0. In addition, it might be desirable to collect a confidence rating in addition to the rating itself. With a confidence range, the rater could communicate not just a point estimate but a full probability density over ratings reflecting subjective certainty. This would be useful for Bayesian inference on the basis of multiple ratings. Such an inference procedure could also include a probabilistic model of each rater’s reliability (e.g., based on past performance). Although negative ratings and confidence ranges will likely prove useful for some of the scales that will come to be used in the system, the first and overall scale for the minimalist system we describe in this section is restricted to positive values, as this is more consistent with this scale’s content and function, as explained below.

Beyond the resolution and range of the scale, we need to decide the content: What evaluation criteria should be captured by the first scale (for which we expect to accumulate the greatest number of ratings)? The scale’s definition must be highly general as any specific choice we make is going to be problematic. Say we defined the scale as measuring the “justification of the claims” of the paper. A user might find a technical paper that is highly justified in its claims less significant than a bold paper that presents a groundbreaking theory and still makes a reasonable case for its claims. Other users will have different priorities. While the proposed system ultimately addresses this issue by enabling multi-dimensional ratings (including open-ended definition of new scales), it still faces a decision for the first scale.

### Desired-impact ratings

We must not put the user in a double bind, where the scale is defined by one criterion, but he or she would prefer to judge by another, in awareness of the real-world consequences of the judgment on the visibility and thus ultimately on the impact of the paper. I therefore propose that the single overall scale should be the “desired impact” for the paper. This describes the actual effect the scale is meant to control and thus avoids the double bind. A user who feels that justification of claims should be the most important criterion will judge desired impact by this criterion. A different user might give more weight to the originality of the ideas put forward. Defining the scale as “desired impact” acknowledges the inherent subjectivity of judging the significance of scientific papers.

Note that the proposed overall scale of “desired impact” is not the only scale that should be used. Other scales will focus explicitly on the justification of the claims of a paper and on other specific evaluative dimensions. Note also that “desired-impact” ratings express *desired*, not *predicted* impact. One might predict great impact for a paper one considers incorrect. But most of us would not *desire* high impact for such a paper. The desired-impact rating enables scientists to judge by their own criteria (including veracity and importance) what impact a paper deserves.

The next question is how desired impact should be expressed numerically. I propose the use of a unit that scientists already understand: the IF. IFs are used in the current system of scientific publishing for evaluating journals. Journal IFs are problematic, especially when they are misinterpreted as measures of the quality of the papers published in a given journal. However, they are widely understood and grounded in the citation success of papers. The IF of a journal is the average number of citations in the present year received by papers published by the journal in the previous 2 years. We can loosely interpret the IF as the average citation success of a paper in the 2 years following the year of its publication.

We define the first scale as “desired impact” in IF units. The IF unit is redefined to apply to a particular paper as measuring the number of citations the paper should receive in the 2 years following the year of its publication, so as to be considered by the user as having received an appropriate amount of attention. Alternatively, we can think of the desired-impact rating as the IF of the hypothetical journal that the paper is deemed appropriate for.

### Rapid rating, optional in-depth reviewing

**Figure 6:**
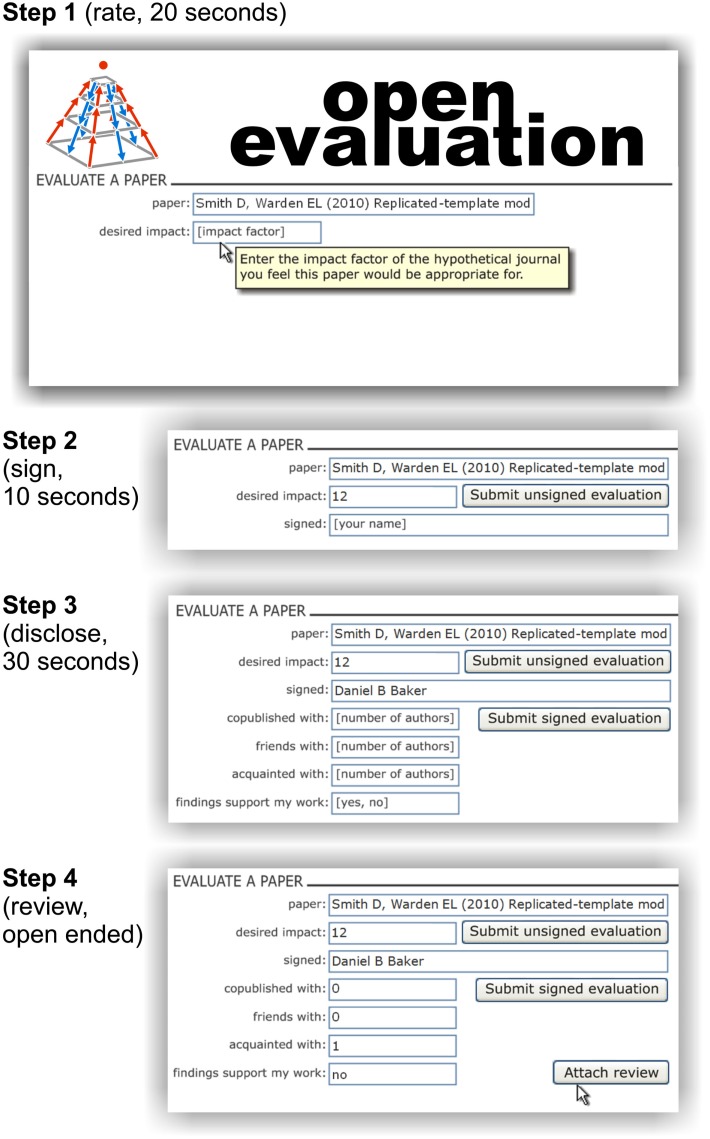
**A minimalist system for accumulating evaluations (ratings and reviews)**. Steps 1–4 illustrate a user’s interaction with an envisioned web interface. (1) Rate: The user selects a paper to evaluate (either by clicking on an evaluation link associated with the paper, or by specifying the paper in the top entry field. The user then enters a single overall numerical rating (desired impact in impact-factor units), whereupon a button labeled “Submit unsigned evaluation” appears (shown in step 2). By clicking this button, the user can submit the overall rating anonymously and terminate the process with a total time-investment of about 20 s. (2) Sign: alternatively, the user can choose to sign the evaluation by entering his or her name, whereupon a button labeled “Submit signed evaluation” appears. By clicking this button, the user can submit the overall rating as a signed evaluation with a total time-investment of about 30 s. (3) Disclose: optionally the user can disclose information on social links to the authors and personal stake in the claims before submission, which might take another 30 s. (4) Review: finally, the user can attach a written review (a txt, doc, or pdf), which can include detailed ratings on multiple scales (in a standard syntax that makes the ratings extractable and enables open-ended definition of new scales), as well as written arguments and figures.

Figure 6 presents a web-interface that provides the functionality for rapidly collecting desired-impact ratings, while “tempting” the user to provide more detailed evaluative evidence. First, the user specifies the paper to be evaluated. This can be done either by clicking a link in PubMed, Google Scholar, or a similar search engine, or by explicitly specifying the paper in the OE interface. The user then enters the desired-impact rating, whereupon a “Submit unsigned evaluation” button appears along with a new field for optional signing of the evaluation. The user can click “Submit unsigned evaluation” and be done in about 20 s, or sign, which might require an additional 10 s.

Signing can utilize existing web identification and authentication technology. It could be automatized using active logins in scientific or non-scientific social networks. For example, Google Gmail, facebook, and Apple iTunes all use such technology. But even if the scientist just signed by name in a text field, the system could work, because all evaluations are public, and identity theft in OE could be righted retrospectively.

The motivation to sign would come from the greater weight certain PEFs will assign to signed evaluations. In some of these PEFs this weight will also depend on an evaluation of the signing scientist. In addition, signing evaluations contributes to the reputation and visibility of the scientist.

After signing, the user has the option to disclose information about social links to the authors and about any personal stake in the results of paper. Within another 30 s, the user can disclose how many of the authors he (1) has co-published with in the past, (2) is friends with, and (3) is acquainted with, and (4) whether the findings reflect positively upon his or her own work. These ratings are made in an honor system. However, since they are public information that can be verified, there is a strong disincentive to misrepresent potential conflicts of interest. As for signing, the positive motivation for disclosing comes from the greater weight some PEFs will assign to ratings, for which this information is available.

Finally, the user is given the option to attach a review. The review can be attached in a suitable format for being read by people and analyzed by PEFs. The existing formats txt, doc, or pdf could initially serve this purpose, although more structured and flexible formats might come to be preferred. A review can contain ratings on multiple scales (which are labeled in a flexible syntax that enables the user to introduce additional scales as needed to capture the quality of the work), along with text and figures. Such a review is an instant citable, mini-publication, providing added motivation for contributing to the process.

## A Specific Plan for a Minimalist Paper Evaluation Function

**Figure 7:**
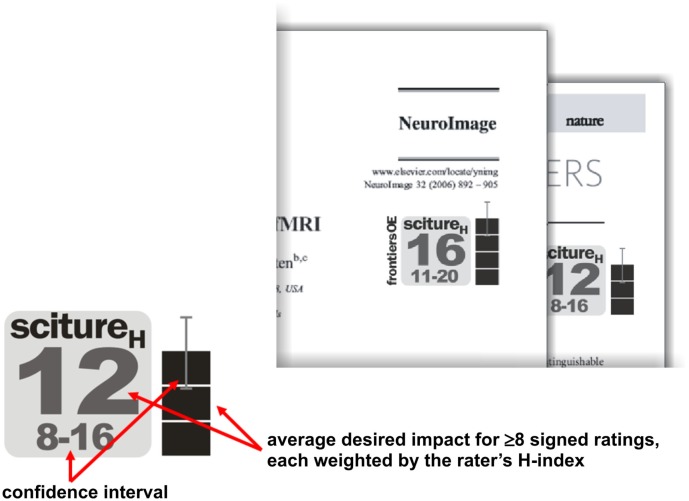
**A minimalist paper evaluation function**. As one possible general-purpose PEF, I suggest the “sciture*_H_*” index, which is an average of at least eight scientists’ desired-impact ratings (in impact-factor units), weighted by the scientists’ *H*-indices. Such an index could serve to provide ongoing open evaluation of papers published under the current system. An icon summarizing the index and its precision (left) could be added to online representations of papers (right), either by the publishers themselves or by independent web-portals providing access to the literature.

The web-based OE system we described above can accumulate the evaluative evidence. However, the evidence still needs to be combined for prioritizing the literature. We have stressed the need for a division of powers between these two components of the evaluation process, and for a plurality of perspectives on the literature in the form of multiple competing PEFs. To make the concept of a PEF more concrete, I propose a blueprint for a general-purpose PEF called “sciture” (Figure 7).

Sciture stands for “scientific citation future.” This particular PEF uses only the desired-impact scale, enabling it to draw from a larger number of ratings than PEFs that combine multiple rating scales (for which we expect to accumulate fewer scientists’ ratings). A paper’s sciture is the impact projected for the paper by the scientists that rated the paper. The sciture is the desired impact in impact-factor units averaged across the scientists who signed their ratings.

There are two variants of the index. The first (simply called sciture) uses an unweighted average of all signed ratings, so as to give raters equal influence. The second is called sciture*_H_* and weights each rating by the rater’s *H*-index (Hirsch, 2005), thus giving more weight to scientists whose own publications have had a greater impact. Note that this index excludes laypeople and young scientists who have never published or whose publications have never been cited. This may be seen as an advantage or a disadvantage and may motivate the definition of alternative PEFs. Note also that the present definition of sciture ignores conflict of interest information. This could be changed in case there were evidence that positive evaluation cliques distort the ratings.

## The Ultimate Goal: Free, Instant, Open-Access Publishing, Peer-to-Peer Editing, and Open Evaluation

### Free instant publishing

Once OE provides the critical evaluation function, papers themselves will no longer strictly need journals in order to become part of the scientific literature. They can be published like the reviews: as digitally signed documents that are instantly publicly available. OE will provide evaluative information for any sufficiently important publication. With OE in place, there is no strong argument for pre-publication review. The binary decision for or against publication will be replaced by graded evaluative evidence, that is summarized by PEFs. Publication on the internet can, thus, be instant and reviews will follow as part of the integrated post-publication process of reception and evaluation.

### Peer-to-peer editing

Peer-to-peer editing can help to get the evaluation process started and to ensure that the initial two to four reviewers are somewhat balanced in terms of biases and expertise. Balance is particularly important in the initial phase, because a small number of negatively biased initial reviews can nip a paper’s OE process in the bud. After publication, the author asks a senior scientist in his or her field to serve as *editor* for the paper. If the senior scientist accepts, an acknowledgment of his or her role as editor will be added to the paper. The editor’s job is to select two to four reviewers and to email them (via an automatic system that uses standard invite texts) with the request to publicly review the paper. If they decline, the editor is to find replacements. However, anyone else is allowed to review the paper as well. In particular, the author may also inform other scientists of the publication and ask them to review the paper. Author- and editor-requested reviews will be marked as such. Reviewers could be asked to state whether or not the review has been requested by an editor or suggested (explicitly or implicitly) by the authors. Requested as well as unrequested reviews can be signed or unsigned. Editors must not have been at the same institution or on any paper with the authors. Reciprocal or within-clique editing is monitored and discouraged. Since the editors are named, PEFs can detect within-clique editing and reviewing and downweight within-clique reviews and within-clique-editor-requested reviews.

### Revisions

If the weight of the criticism in the accumulated reviews and the importance of the paper justify it, the authors have the option to revise their paper. The revision will then be the first thing the reader sees when accessing the paper and the authors’ response to the reviews may render the criticism obsolete. However, the history of revisions of the paper, starting from the original publication will remain accessible in perpetuity.

### Reviews as mini-publications

**Figure 8:**
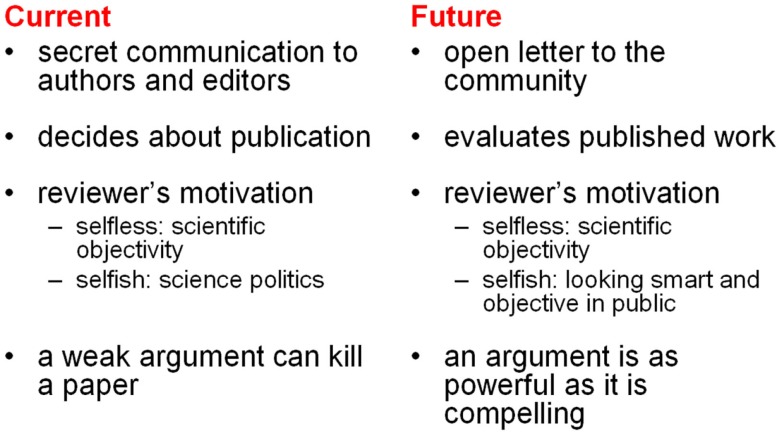
**The nature of a review in the current and future systems**. This juxtaposition of the key features of a review in the current and the future system points to some essential changes in scientific culture that the transition will entail.

Reviews will no longer be secret communications deciding about publication. They will be open letters to the community with numerical quality ratings that will influence a paper’s visibility on web-portals. The quality and quantity of signed reviews written by a given scientist will be one of the determinants of his or her status. This will greatly enhance the motivation to participate in the evaluation process. With a general OE system in place, reviewing activity can be analyzed with the same methods used to analyze other publication activity. Figure 8 contrasts the nature of a review in the current and in the proposed system. Through transparency, the proposed system replaces the unhealthy incentives of the current system (i.e., to minimize the time spent reviewing and to exert political influence) by healthy ones (i.e., to contribute objective and reasonable evaluations so as to build one’s reputation).

### A different culture of science

Open evaluation goes hand in hand with a new culture of science. This culture will be more open, transparent, and community controlled than the current one. We will define ourselves as scientists not only by our primary research papers, but also by our signed reviews, and by the prior work we value through our public signed ratings. The current clear distinction between the two senses of “review” (as an evaluation of a particular paper and as a summary and reflection upon a set of prior papers) will blur. Reviews will be the meta-publications that evaluate and integrate the literature and enable us as a community to form coherent views and overviews of exploding and increasingly specialized literatures. Evaluation of scientific work and distillation of the key insights are at the heart of the entire endeavor of science. The scientific community will therefore take on the challenge of designing and continually improving the evaluation system. This includes design of the human-computer interfaces, design of the web-mediated interactions between humans, and design of artificial-intelligence components that will help evaluate and integrate our insights. Designing the OE system will lead us to the ultimate challenge: to design the collective cognitive process by which science, globally connected through the web, constructs our view of the world, and ourselves.

## Discussion

The discussion is structured by critical questions that I have encountered when discussing this proposal.

### If peer review occurs only post-publication, won’t the literature be swamped with low-quality papers that are never evaluated?

Yes, but that’s not a problem. Peer review currently serves as a barrier to entry into the literature, serving to maintain a certain quality standard. Removing this barrier might seem dangerous in that it might open the gates to a flood of low-quality papers. In other notable proposals of public peer review, pre-publication review therefore still plays a role (e.g., Bachmann, 2011; Kravitz and Baker, 2011; Pöschl, 2012; Sandewall, 2012; all in this collection). Here, we argue that pre-publication peer review is not needed or desirable. Peer evaluation will help us select what to read, but it will not prevent us from reading papers that have not (yet) been evaluated.

#### Minimal formal barriers to publication

For a paper to become a citable and permanently archived publication, the authors’ identities need to be verified. In addition, a restriction could be placed on the volume of work per author (e.g., 12 papers per year). This would help prevent computer-generated content from being submitted. Beyond these formal restrictions, authors will be aware that low-quality publications will damage their reputations. Scientific papers require minimal storage (compared to other cultural products, such as movies) and their number is small per capita of the population and year. Although the total storage required will be substantial, our technology can handle it.

#### Only published papers can be publicly evaluated

Peer evaluation cannot be truly open (i.e., public) unless the paper is publicly available (i.e., “published”). A public peer review, thus, is post-publication by definition. A pre-publication stage would be merely a matter of labeling published papers as either “under review” or “reviewed” (i.e., “properly published”). However, OE is to be ongoing and incremental, and the evaluative signal continuous and multidimensional. Labeling already published papers as “reviewed” or “properly published” at some stage merely amounts to imposing an arbitrary threshold on some PEF. There is no clear motivation, thus, for dividing OE into two stages.

#### The twilight zone of unevaluated papers

Some published papers will never get a single review or rating; this is not a problem. There will be a new twilight zone of published, citable, but unevaluated papers. As readers, we do not mind this, because twilight papers will not come to our attention unless we explicitly search for them. As authors whose work remains in the twilight, we will learn that we need to connect better with peers through conferences, conversations, and high-quality work, to earn enough respect to find an initial audience, and a peer-to-peer editor. In case we are too far ahead of our peers to be understood, our twilight publications might be discovered later on. The future system will thus provide a mechanism for publication of science that defies the dominant scientific paradigm, is unpopular for other reasons, or simply difficult to understand. However, there is no instant mechanism for distinguishing the bad from the brilliant, but misunderstood. It is therefore necessary to provide permanent access to both, and unavoidable that a proportion of the literature will receive little attention and no proper evaluation.

### What if there are too few evaluations for a paper?

#### Papers with less than eight ratings will come with large error bars, or without error bars

Many papers will receive some evaluations, but not enough for reliable averages. These papers are under evaluated as are all papers in the current system. In the proposed system, however, the lack of reliable evaluation will be reflected in the absence (or large range) of the error bars on the overall score from a given PEF.

#### Important papers will be broadly and deeply evaluated

Important work will eventually be read, rated, and reviewed. Because a scientist’s time is a limited resource, broad and deep evaluation can only be achieved for a subset of papers. Broad evaluation means that many scientists from different fields participate in the evaluation. Deep evaluation means that experts in the field provide in-depth evaluations and commentary on the details. To the extent that an initial set of reviews brings more attention to a paper, it will tend to be more broadly and deeply evaluated. This selective and recurrent allocation of the field’s attention is a key feature of the proposed system. Selective recurrent rating and reviewing ensures that we have a reliable evaluation before raising a paper to global visibility within science and before bringing it to the attention of the general public.

### How can scientists be motivated to submit reviews in an open peer-review system?

#### Scientists accept requests to review papers in the current system – this will not change

In the current system, scientists are approached by editors and asked to review new papers. They regularly comply. In the new system, they will be approached similarly often through peer-to-peer editing with the same request – only the reviews will be public. There is some evidence that potential reviewers are more likely to decline to review when they are told that their name will be revealed to the authors (van Rooyen et al., 1999) or that their review might be publicly posted (van Rooyen et al., 2010). This reflects the culture of the current system, in which the reviewer expects no benefits, except the opportunity to read new work, to help improve it, and to contribute to the publication decision. In this context, removing anonymity appears to have no upside and could pose a threat. In the future system, however, reviews will be mini-publications that bring substantial benefits to the reviewer.

#### The motivation to review a paper is greater if the review is an open letter to the community

The fact that reviews are public makes reviewing a more meaningful and motivating activity. In terms of power, the reviewer loses and gains in the transition to the proposed system: The reviewer loses the power to prevent or promote the publication of a paper by means of a secret review. The reviewer gains the power to speak to the whole community about the merits and shortcomings of the paper, thus building his or her reputation. The power lost is the secretive and political kind of power, which corrupts. The power gained is the open and objective kind of power that motivates constructive critical argument.

#### Signed reviews will be citable mini-publications contributing to a scientist’s reputation

Reviews will be citable publications in their own right. This will motivate reviewers in terms of quality and quantity. Moreover, reviews can themselves be subject to second-order peer evaluation. Reviewing will gain in importance, because it is critical to the hierarchical organization of an exploding body of knowledge. Reviewing will therefore become a scientific activity that is more publicly valued and formally acknowledged than it currently is. Conversely, the absence of a contribution to OE will reflect negatively on a scientist. These factors will increase the motivation to participate in the evaluation process.

### Will signed reviews not be positively biased?

Signed reviews might indeed be affected by a positive bias (Walsh et al., 2000). Reviewers might want to please particular authors or groups (specific bias), or they might want to be perceived as nice people (general bias). However, this is not a problem for four reasons:

(1) *Reviewers are motivated to minimize bias when they sign their reviews: their reputation is on the line*: The perception of a specific bias attributable to the reviewer’s academic or social connections or to a self-serving preference for certain theories would seriously threaten the reviewer’s reputation. To a lesser extent, a reviewer who signs will also be motivated to minimize general positive bias, which might result from the desire to appear to be a nice person. A general “niceness bias” would suggest that the reviewer is undiscerning and thus fails to contribute critical judgment.
(2) *A general positive bias will not compromise the assessment of the relative merit of different papers*: Even if each reviewer were affected by niceness bias to some degree, the relative merit of different papers could still be judged. The extreme scenario would be an endorsement culture, where only positive reviews are ever signed. This is comparable to reference letters, which are meant to help evaluate people’s abilities. Reference letters are affected by massive positive biases, but still serve their purpose. Even if all signed evaluations were positive endorsements, the number of endorsements, the numerical ratings, and the level of enthusiasm of the positive reviews would still offer valuable measures of the community’s appreciation of a paper.
(3) *Biases of signing reviewers can be measured and corrected for by PEFs*: For a given reviewer, the set of signed reviews written and the distribution of signed numerical ratings given are public information. PEFs could therefore estimate and remove biases. For example, each reviewer’s ratings could be converted to percentiles, reflecting the relative rating in comparison to the other studies reviewed by the same person. In addition, a reviewer’s general bias could be assessed by comparing each of his or her ratings to the mean of the other reviewer ratings across all papers reviewed. As for specific biases reflecting academic or social connections or preferences for particular theories, these too could be automatically assessed. The suggested minimalist OE system already includes optional disclosure of information that might suggest biases (i.e., collaborative or social connections to the authors and a personal stake in the results). When the reviewer does not volunteer such information, his or her ratings could be downweighted preemptively. Moreover, analyses of social and academic networks and of published papers could be used to estimate the probability of a conflict of interest. Again, PEFs could use such estimates to adjust the weight assigned to a reviewer’s ratings.
(4) *Signing reviews is optional*: If a reviewer feels timid about signing a critical argument, he or she can contribute the argument without signing the review. Unsigned reviews and ratings might be given less weight in some PEFs. However, other reviewers who invest enough time in the paper to read the previous reviews may pick up the argument if it is compelling in their own signed reviews. Note that there is no need for an ethical requirement that a single scientist either sign or not sign all reviews. Instead signed and unsigned reviews serve complementary positive roles and the choice between them motivates a richer and freer exchange of arguments.

### How can reviews and reviewers be evaluated?

A key decision in the design of a PEF is how to weight the ratings of different reviewers. First, signed ratings can be weighted by evaluations of the reviewers who gave them. In the sciture*_H_* index suggested above, each rating is weighted by the reviewer’s *H*-index. Alternative indices of the reviewer’s general scientific performance could equally be used (Kreiman and Maunsell, 2011 in this collection). However, it might be preferable to evaluate a reviewer’s performance at the specific task of reviewing, e.g., by estimating the predictive power of their past reviews or by relying on meta-reviews of their past reviews (see Wicherts et al., 2012; in this collection). Second, without evaluating the reviewer, we can directly evaluate a given rating or review to determine its weight. Some ways of evaluating a particular review or rating and the overall performance of a reviewer are as follows.

#### Reviews and ratings can be evaluated by meta-reviews and meta-ratings

A review is a mini-publication that evaluates another publication. That other publication can be another review. This simple mechanism enables scientists to rate and review ratings and reviews. It can also serve as a mechanism for authors to respond to reviews. PEFs exploiting meta-ratings can recursively compute the weights, employing heuristics that prevent meta-raters from neutralizing substantial judgments. For example, a PEF might ignore unsigned meta-ratings and meta-ratings signed by one of the authors of the original paper.

#### Reviews can be evaluated through reviewer self-report of relevant information

Reviewers can self-report numerical information relevant to weighting their reviews. This information would be part of the ratings block in the review text. In the minimalist OE system described above, reviewers can disclose personal links to the authors of the paper and a personal stake in the claims. In addition, reviewers could self-report a confidence interval for their ratings. Self-report of confidence would enable optimal statistical combination of multiple reviewers’ contributions in PEFs. Reviewers would have an incentive to accurately assess their own confidence because an error with high self-reported confidence would have a stronger impact on their reputation. Another potentially helpful piece of information is a reviewer’s time-investment in the review. A judgment based on several days of reading the paper, thinking about it, and further researching key issues might be given greater weight than a judgment made in passing. Self-report of time-investment would be an honor system. However, time-investment ratings could be summed to check a reviewer’s total claimed time-investment for plausibility. If the total time-investment exceeded 8 h per day, the reviewer could be discredited or downweighted. A reviewer’s total number of reviews (in a given year) and total time spent reviewing could also be used to limit a single person’s influence.

#### Reviewers can be evaluated by the predictive power of their reviews

A reviewer who signs a review or rating links a little piece of his or her reputation to a paper. This is a gamble. Say the review was positive. If the paper stands the test of time, then the reviewer’s reputation rises a little. If the paper becomes discredited, the reviewer’s reputation falls. Since every scientist rates many papers, a single erroneous judgment will not have a large effect. A reviewer’s performance on a given evaluation can be estimated as a function of the existing evaluations at the time of submission of the evaluation and the evaluations accumulated up to the present moment: Performance could be judged as high if the reviewer’s judgment stands the test of time, and especially high if this evaluation was made early and/or diverged from existing evaluations when it was entered. This criterion can be formalized in an information-theoretic framework.

The OE system will enable scientists to make visible contributions by evaluating others’ work. As a result, reviewing will be a competitive, public activity, that strongly impacts one’s reputation as a scientist. Some scientists will contribute to the evaluation more than others. In fact, the system would enable some scientists to specialize in this particular form of meta-science. The system will fundamentally change the way science progresses: scientists will want to attach their reputations to the developments they truly believe in. Looking wisely ahead with deep intuition will be rewarded over following shallow trends.

### Will instant publishing not destroy the constructive process of review and revision?

#### No, revisions will still be possible in the proposed system, and they will often include improvements made in response to reviews

A revision will take precedence over the original version of the paper in that it will be the version most visibly presented to readers. However, the entire history of the paper, including the original version, all revisions, and all evaluative meta-information will remain openly accessible and separately citable in perpetuity. The authors have no right or ability to remove this record.

If the authors decide to submit a revision of their paper, the revision will require re-review (as is the case in the current system for major revisions). The ratings and reviews of the original paper will not automatically transfer to the revision. If the revision is important to the field, it will be re-evaluated by enough scientists (likely including some of the original reviewers). If the revision is less important, it will not be as broadly and deeply evaluated as the original version, but can still serve to provide the most up-to-date version of the paper and address the reviews of the original.

The authors are free to refuse to revise their paper if other projects are of greater importance to them. When the authors disagree with reviews, they can publish responses to the reviews (as meta-reviews), which may contain further experimental results, along with ratings of the reviews. PEFs may utilize higher-order reviews in weighting the ratings of the first-order reviews. Responses to reviews are simply reviews referring to other reviews, thus utilizing the same infrastructure as reviews of papers and meta-reviews contributed by other scientists. Author responses to reviews and will provide an important function complementary to that of a revision.

#### Scientists will be highly motivated to seek informal feedback before publication of the original paper

A paper, once published, can never be erased from the crystallized record of scientific history. Moreover, the attention the community grants to a new paper upon publication so as to evaluate it may not be reduplicated for a revision. This creates a strong motivation for scientists to publish only work they can stand by in the long run. Scientists will therefore seek informal constructive criticism before initial publication to a greater degree than currently. For example, in addition to presenting the project at a conference they may post the paper on a blog or share it with selected researchers by email a few weeks before publication. This informal round of review and revision will reduce the noise in the crystallized record.

### Can alternative metrics, including usage statistics, social-network information, and link-based importance indices, serve to prioritize the literature?

Yes, alternative metrics derived from usage statistics, from links, and from the social web will play an increasing role in steering the attention of both the general public and the scientific community (Neylon and Wu, 2009; Priem and Hemminger, 2010; see also this collection: Birukou et al., 2011; Walther and van den Bosch, 2012; Zimmermann et al., 2012). However, evaluating science also requires conscious judgment by experts. In addition to the informal and fleeting buzz of the social web, we therefore need a system to collect and analyze explicit peer reviews and ratings.

Algorithms like PageRank (used by Google to prioritize search results) can provide overall importance indices, and can be modified to rely more heavily on some links (e.g., citations from scientific papers) than others. In usage and link-based importance indices, however, positive and negative attention adds to the visibility of the content. Explicit judgments, such as the “desired-impact” rating suggested above, provide a complementary signal that will be important in science. In contrast perhaps to other domains like art and entertainment, science will always rely on explicit peer judgment.

### Can research blogging serve the function of open peer review, and perhaps even of scientific publishing in general?

**Figure 9:**
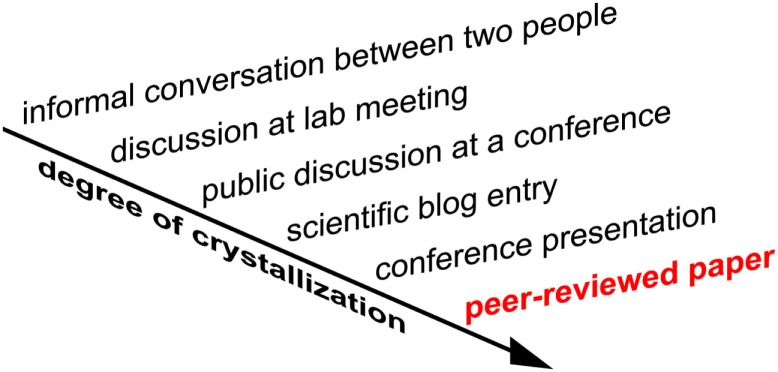
**Degrees of crystallization of scientific communications**. Scientific communications, from informal conversations to peer-reviewed papers, span a wide range of degrees of crystallization. Crystallization increases with the number of people in whose memory the communication is stored and with the reliability, permanence, and citability of computer-based storage. The most significant scientific communications deserve lasting accessibility and citability. They form the historical memory of science. The peer-reviewed paper, thus, will continue to play a pivotal role in science, even as more fleeting online communications gain in importance.

Research blogging fills an important gap: between informal discussions and formal publications (Harnad, 1990). Unlike a private informal discussion, a blog is publicly accessible. Unlike a scientific paper, a blog post can be altered or removed from public access. Blog posts are also often anonymous, whereas papers are signed and author-authenticated. These more fluid properties of blogs make for their unique contribution to scientific culture. However, the very fluidity of blogs also makes them inadequate as the sole vessel of scientific publishing. In particular, blogging lacks the quality of “crystallization” (Figure 9). A scientific publication needs to be crystallized in the sense that it is a constant historical record that can be accessed permanently and therefore cited.

**Figure 10:**
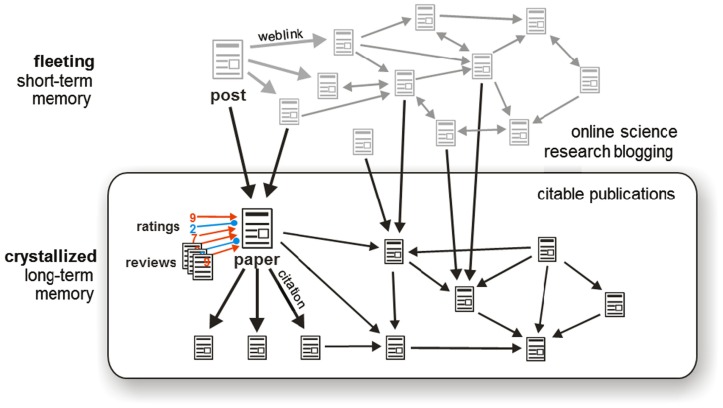
**Fleeting online science and the crystallized scientific record**. Much of online science is fleeting. For example, a link to a blog post becomes obsolete when the owner removes the post. This is as it should be. Research blogs serve as science’s short-term memory. However, science also needs a long-term memory, a crystallized and permanently citable historical record. This function is served by the peer-reviewed literature. Note that fleeting online science and the crystallized record interact intensely as bloggers refer to papers and blogs inspire new studies that later become part of the scientific record. However, while blogs link to other blogs (gray arrows) and cite papers (black downward arrows), scientific papers mainly cite other scientific papers (black arrows), because links to online science are less dependable in the long term.

Blogs are science’s short-term memory (Figure 10). They enable more intuitive and divergent reasoning. The crystallized literature is science’s long-term memory, which enables more analytical and convergent reasoning. Crystallized scientific publications include papers and reviews. Reviews are crystallized publications that serve mainly to evaluate one or several other crystallized publications. Crystallized publications are digitally authenticated documents that reference other scientific publications.

The web’s equivalent of a citation is a link. Links are versatile and fast, but there is no mechanism to ensure that they will continue to work in perpetuity. In fact, such a mechanism would rob the web of a key feature: plasticity. While the web world of blogs is fast and flexible, it is also fleeting and this is a good thing. As a complement to the web, however, we need a crystallized scientific record. Links here are citations of papers identified by digital object identifiers, which are guaranteed to be maintained in perpetuity. Links crossing the boundary between these two worlds are desirable. Scientific posts (i.e., a web document such as a blog post) will use web-links to other non-crystallized resources and in addition they will cite the crystallized record. Conversely, scientific papers (i.e., crystallized publications) will rely on citations to ground themselves in the crystallized scientific record and can additionally utilize web-links, with the understanding that these may become defunct.

### What is the role of publishing companies and journals in the proposed system?

This proposal affirms the importance of the scientific paper and the process of peer review as essential elements of scientific publishing. The current function of the journal in administrating peer review, selecting content, and providing access to related papers in context will be more fluidly served by web-portals that present a portion of the literature, prioritized by PEFs. The future system will be designed by scientists, independent of publishing companies. This reflects the fact that the key functions of access and evaluation can be served at a higher level of quality and at lower costs than in the current system.

However, for-profit scientific publishers will have new opportunities to offer services that will legitimately contribute to science and society. The publication and review of specialized scientific papers might no longer depend on for-profit publishers, but their services can contribute to communicating the most important scientific findings beyond the confines of a highly specialized scientific audience. As an example of this challenge, let’s consider the role currently played by the high-prestige publications *Nature* and *Science*.

*Nature* and *Science* strive to reach a broad scientific audience with groundbreaking new science. They succeed in publishing many important papers. However, they use classical peer review, so their evaluation mechanism suffers from non-transparency (secret review) and from a lack of evaluative evidence (2–5 reviews). As a result, *Science* and *Nature*, despite having the highest standard in the industry, do not quite live up to their promise of publishing only groundbreaking work. Conversely, they miss out on groundbreaking work published elsewhere (because it was either not submitted to them or rejected by them). In addition, primary research papers in *Nature* and *Science* do not typically succeed at communicating their results to a broad audience.

In the future, a for-profit publisher could utilize the OE system, develop its own PEF for selecting content, and produce a high-prestige publication that fully succeeds (1) at presenting only groundbreaking science and (2) at communicating it to a broader audience. The content of such a general science magazine would not be primary reports of new scientific findings. Rather the publisher would select independently published studies that have turned out to be groundbreaking, relying on the broader, deeper, and more reliable evidence from OE. The original authors would then be invited to write a piece communicating the science more broadly (cf. the “Focused Review” format of *Frontiers*). Since the scientific validity and significance has already been established, the publisher’s role would be to ensure readability and didactic quality of text and visuals. Copy editing and professional artwork and layout, as provided by publishing companies, are non-essential for primary research reports, but valuable for the broader communication of groundbreaking findings.

### How can we realistically transition to the proposed system?

Transitioning to a radically different system is difficult. Clearing the slate and starting from scratch, i.e., *revolution*, is often politically and logistically unrealistic. In addition, no matter how brilliant and detailed our vision for the future system, it is bound to fall short of anticipating all complications encountered during implementation. Our vision might even be fundamentally flawed, in which case revolutionary change would be a catastrophic mistake.

Transitioning through *evolution*, on the other hand, is not always possible. The present system may be stuck in a local optimum, where any small changes worsen the situation. This could be among the reasons for the persistence of the current system of scientific publishing. Senior scientists and editors who appreciate the subtle checks and balances of the current system may feel that suggestions for change are naive and would not improve the situation.

Fortunately, there is a continuous path toward fundamental change of the scientific publishing system. To make change, we need to open up not only access, but also evaluation. Access and evaluation are the two major functions a publishing system must provide. With OA on the rise, evaluation, i.e., the stamp of approval implicit to acceptance of a paper in a journal of a given level of prestige, is the essential product the scientific publishers are selling today. Once scientists take on the challenge of envisioning, implementing, and using an independent and general OE system, change is underway.

An independently built OE system can evaluate the entire literature, including papers published under the current system, which appear in traditional journals. The tipping point is reached when the evaluations provided by the OE system are perceived as more reliable and authoritative than journal prestige as an indication of a new paper’s quality. At this point, scientists will no longer be dependent on journals to publish their work.

The key challenge therefore is for the scientific community to converge on a vision for OE. This will require alternative proposals to be explored in detailed papers and to be widely discussed. We hope that the collection of visions presented in this Research Topic will contribute to this process.

### Who is responsible for designing and continually improving the publishing system?

It’s up to scientists to design and continually improve the future publishing system. Providing access and evaluation of the literature is properly construed as a key methodological challenge for science. Science tackles other difficult methodological challenges by means of methodological studies and a literature documenting the results. We also need a literature, both theoretical and empirical, exploring methods for OE.

So far scientists have largely left the design and justification of the evaluation process to journals and publishing companies. However, the evaluation system is a core component of science itself. It determines the confidence we can have in scientific findings. It steers the attention of the scientific community and affects public policy decisions. The evaluation system, therefore, must be designed by scientists. The behavioral, cognitive, computational, and brain sciences are best prepared to take on this task, which will involve social and psychological considerations, software design, and modeling of the network of scientific papers and their interrelationships. We need a literature that illuminates how we can bring science and statistics to the evaluation process.

The larger challenge is to design the collective cognition of the scientific community and its interaction with web-based artificial intelligence. OE is a core component of this collective cognitive system. Designing OE requires us to study (1) the individual scientist’s motivation, cognition, and interaction with web-based human-computer interfaces, (2) the consequences of enabling different forms of individual influence on the system, (3) the dynamics of the entire system as a social network, (4) mechanisms for combining evaluations from many individual scientists so as to prioritize the literature, (5) the network of papers (nodes) and citations (links) and potential automatic inference methods (e.g., Bayesian belief propagation) that can be applied to this network to assess the validity of the claims in the context of their interrelationships.

### Shouldn’t we strive for an even more radical vision of collaborative science?

Yes, we should. Web collaboration is bound to revolutionize the way science is done (Nielsen, 2009, 2011). Scientific teams collaborating on a problem will be distributed around the world and as the process becomes transparent throughout, traditional divisions will blur and might well evaporate. These divisions include the temporal division between the stages of doing the science, of publishing it, and of review and reception; the division of communications between collaborative communication among the team and publication of the results; and the social division between team members (i.e., coauthors) and the audience of scientists exposed to the results. However, even when this dream has become a reality, we will still need a permanent record of scientific papers and explicit peer judgments. The present proposal focuses on this permanent record, but fits well into a larger vision of fluid, open, collaborative science on the web.

## Conflict of Interest Statement

The author declares that the research was conducted in the absence of any commercial or financial relationships that could be construed as a potential conflict of interest.

## Footnotes

^1^http://researchblogging.org/

^2^http://www.plos.org/

^3^http://www.plosone.org/home.action

^4^http://frontiersin.org/

^5^http://www.facultyof1000.com/

^6^http://en.wikipedia.org/wiki/Public-key_cryptography

^7^http://code.google.com/p/gpeerreview/
